# Diet of the brown bear in Himalaya: Combining classical and molecular genetic techniques

**DOI:** 10.1371/journal.pone.0225698

**Published:** 2019-12-26

**Authors:** Muhammad Ali Nawaz, Alice Valentini, Noor Kamal Khan, Christian Miquel, Pierre Taberlet, Jon E. Swenson

**Affiliations:** 1 Department of Animal Sciences, Quaid-i-Azam University, Islamabad, Pakistan; 2 Department of Ecology and Natural Resource Management, Norwegian University of Life Sciences, Norway; 3 Himalayan Wildlife Foundation, Islamabad, Pakistan; 4 Norwegian Institute for Nature Research, Trondheim, Norway; 5 Laboratoire d'Ecologie Alpine, Université Joseph Fourier, France; 6 Dipartimento di Ecologia e Sviluppo Economico Sostenibile, Università degli Studi della Tuscia, Viterbo, Italy; Institute of Zoology, CHINA

## Abstract

The ecological requirements of brown bears are poorly known in the Himalaya region, which complicates conservation efforts. We documented the diet of the Himalayan brown bear (*Ursus arctos isabellinus*) by combining classical scat analysis and a newly developed molecular genetic technique (the *trn*L approach), in Deosai National Park, Pakistan. Brown bears consumed over 50 plant species, invertebrates, ungulates, and several rodents. Eight plant families; Poaceae, Polygonaceae, Cyperaceae, Apiaceae, Asteraceae, Caryophyllaceae, Lamiaceae, and Rubiaceae were commonly eaten with graminoids comprising the bulk of the diet. Golden marmots comprised the major mammalian biomass in the park, and were also the main meat source for bears. Animal matter, making 36% of dietary content, contributed half of the digestible energy, due to its higher nutritious value. We did not find a significant temporal pattern in diet, perhaps because the availability of the major diet (graminoids) did not change over the foraging period. Male brown bears were more carnivorous than females, probably because of their larger size, which requires higher energy and also makes them more efficient in capturing marmots. Frequencies of three plant species were also significantly higher in male brown bears; *Bistorta affinis*, *Carex diluta*, and *Carex* sp. Diet of the brown bear differed significantly between the park and surrounding valleys. In valleys, diet consisted predominantly of graminoids and crops, whereas the park provided more nutritious and diverse foodThe estimated digestible energy available to brown bears in Deosai was the lowest documented among brown bear populations, due to the lack of fruits and a relatively lower meat content. The low nutritious diet and high cost of metabolism in a high-altitude environment, probably explains the very low reproductive potential of this population.

## Introduction

Knowledge of diet and foraging behaviour is important in the understanding of animal ecology and evolution, especially when they focus on broader nutritional interactions from an ecological perspective[1,2]. Such studies help identify key environmental resources required by a species, and thus enhance the understanding of habitat preferences and provide a knowledge base for successful management and conservation of wildlife populations. Due to growing recognition and methodological advancements, our understanding of nutritional ecology of bears has advanced significantly in the past two decades [3]. However, most diet studies of brown bear have been conducted in North America (e.g. [4–6]) or in Europe (e.g. [7,8]). The brown bear (*Ursus arctos*) is an opportunistic omnivore with a wide geographical distribution [9] and utilizes food according to local availability [10,11]. Therefore, the knowledge of brown bear diet from North America or Europe cannot be generalized for other geographical locations. There is limited information on the diet of brown bears in Asia [12–14] and in particular, no studies exist from the Himalaya, Karakoram and Hindu Kush ranges in South Asia. In order to plan and implement effective conservation programs for brown bears in Himalaya, a sound knowledge of nutritional ecology is essential [15].

The Himalayan brown bear (*U*. *a*. *isabellinus*) is distributed in small populations over the Himalaya, Karakoram, Hindu Kush, Pamir, western Kunlun Shan, and Tian Shan ranges in southern Asia [16]. They are highly threatened throughout their range due to poaching, habitat loss and fragmentation, yet their ecological requirements are generally not known. The reproductive rate is a critical factor in population viability of bears, because they have the slowest reproductive rate of any terrestrial mammal[17]. A long term monitoring study (1993–2006) of brown bears in Deosai National Park (DNP), Pakistan documented extremely low reproductive performance, due to late age of first reproduction (8.25 years), a long reproductive interval (5.7 years), and a small litter size (1.33) [18]. This study showed that the brown bear population in DNP is the least productive studied population in the world. A positive relationship between the diet of bears and their reproductive performance has been documented in a wide range of studies [19–23]. In North America, >90% of the variation in age of first reproduction was explained by vegetational productivity[24]. Autumn body mass, which is dependent on local food conditions, is an important indicator of reproductive output in bears [21–23].

The aim of our study was to document the diet of the brown bear in DNP in relation to its availability and contribution to energy assimilation. For this purpose; we assessed the availability of food resources, determined consumption of food by brown bears by combining classical scat analysis and molecular genetic techniques, and calculated the nutritional value of ingested food and its contribution to energy assimilation. We compared digestible energy (per unit of ingested food acquired) by brown bears in DNP with other brown bears in Asia and elsewhere.

We also investigated temporal and habitat effects, because seasonal and habitat variation in diet has been reported for brown bears [5,8,10,25,26]. Mattson suggested that gender-related nutritional needs may result in sex differences in diet [27]. Though not consistent in all studies [28–30], male bears often eat more meat than females [30–34]. We tested if sex-related differences exist in the selection of plant species or in overall diet items.

## Materials and methods

### Study area

The DNP occupies about 1800 km^2^ of an alpine plateau in the western Himalayas and is managed administratively by the Northern Areas Forest and Wildlife Department, Northern Areas, Pakistan. It is a typical high-altitude ecosystem, with mean daily temperatures ranging from –20°C to 12°C, and annual precipitation varying between 510 mm and 750 mm. It is above the timberline and vegetation is predominately herbaceous perennials, grasses and sedges. There are four kinds of habitats represented in the park; marshy, grassy, stony and rocky [35]. Marshy habitat is dominated by *Poa* and *Carex* spp., with some herbaceous plants. Grassy habitat is dominated by the Poaceace family, and stony habitat has great variety of herbaceous flowering plants. Rocky habitat is generally devoid of vegetation. Marshy habitats contribute most to the forage production, followed by grassy and stony vegetation habitats, whereas rocky areas are unproductive. The surrounding valleys have habitats distinct from the park (coniferous forest, shrubs, rocky and grassy slopes).

The park is covered by snow most of the year (generally October-May). Therefore, brown bears, which usually den in the surrounding valleys, come to DNP in June and leave in early October, when the snow returns. Most scat samples were collected in the park, but 43 were collected from the adjacent valleys, which provided insight into the diet of brown bears there.

### Sample collection

We searched for bear feces throughout the study area from June to early October, during 2004–2005 and 2007. We divided the study area into five blocks, and searched each block for scats each year, covering most of DNP (see details in [36]). In addition, the DNP field staff collected scats during their normal patrolling of the park. For most of fecal samples, the date and location (Geographic latitude/longitude) were recorded using a Global Positioning System (GPS) receiver (Garmin 12XL). Scats were air dried and stored in polythene bags for analysis in the lab.

Samples for genetic analysis (1 cm^3^) were collected in 20-ml plastic bottles with a stick of wood. Bottles were then filled with 95% alcohol to preserve the samples until DNA extraction. We also collected 112 plant specimens from Deosai and preserved them in silica gel. These plants were identified by taxonomists from the University of Karachi, Karachi Pakistan Museum of Natural History, Islamabad, and Quaid-i-Azam University, Islamabad.

### Food availability

A total of 460 plant species have been identified from DNP, including 45 families and over 130 genera [37]. Asteraceae is the largest family, comprising 93 species, followed by Poaceae, 42 and Cyperaceae, 31. Other large families include Rosaceae, Schrophulariaceae, Polygonaceae and Fabaceae, with 25, 24, 23, and 22 species, respectively. For this study, we collected 112 plant species that were likely bear foods (based on field observations), 91 of those could be sequenced for whole chloroplast *trn*L (UAA [38], and 73 with identification at the species level were added to GenBank (accession numbers EU326032-EU326103, [39]. This reference database was used to identify plant sequences obtained from brown bear feces (see details below).

Slate-colored snow trout (*Diptychus maculatus*) and fleshy-mouthed snow trout (*Ptychobarbus conirostris*) are the only two fish species found in DNP [40] and were relatively abundant (pers. obs.). The ground-dwelling invertebrate fauna in DNP was sampled in 1999 [41]. It consisted of four classes, 13 orders and 102 determined families. Based on dry mass, five families dominated; Acrididae (24.6%), Tenebrionidae (13.7%), Lycosidae (11.7%), Carabidae (10.9%), and Anthrophoridae (9.4%).

Himalayan ibex (*Capra ibex sibrica*) and musk deer (*Moschus moschiferus*) occur in and around DNP, whereas the formerly common Ladakh urial (*Ovis orientalis vignei*) is locally extinct. We used field observations of the park staff, and surveys conduced in 2005 to estimate the populations of these ungulates.

Woods et al. recorded seven small mammal species in DNP (*Alticola argentatus*, *Sicista concolar*, *Sorex thibetanus*, *Hyperacrius fertilis*, *Marmota caudata* (golden marmot), *Mustela erminea*, *Ochotona roylei*) and provided their relative numbers [40]. *H*. *fertilis* is the most abundant species, followed by *M caudata* and *A*. *argentatus*. However all of these species are small (20–200 g weight), except for the golden marmot, which weighs ca. 3.5 kg [42] and comprises 97% of the biomass of rodents in DNP. From this and a study of activity patterns, which documented that bears dig out marmot colonies [43], we expected that marmots would be an important component of brown bear diet. Thus, we estimated the density of marmots in the park by walking 500-m wide line transects in 2004–2006. We walked along randomly placed transects, counted marmot colonies within the transects, and marked our routes with a GPS receiver. We plotted the routes of all transects on a map of the study area in ArcGIS (ESRI Inc. 2006) and calculated lengths. Colony densities were calculated from transect areas and multiplied by the average size of a social group (4.0 ± 0.22, [42]) to estimate marmot densities. In 2004, 14 transects were subdivided into habitat types, to calculate relative densities by habitat type. At each colony, we noted whether it had been dug out by brown bears to estimate accumulated brown bear impact on marmots.

### Diet composition

Two life forms of plants, graminoids and herbs, dominate in Deosai and in the bears’ diet. Therefore, it was difficult to differentiate diet components in scats on the basis of morphology. To overcome this limitation, we combined the classical scat analysis and a newly developed molecular technique (*trn*L approach, [44]) to identify diet components to a finer detail.

### Scat analysis

We measured the volume of all scats before analysis by water displacement in a 2-l beaker. Scats were soaked and washed through a 0.8-mm mesh (same size used by Dahle [8]). We selected three sub-samples from this homogenized mixture and analyzed them in a petri dish under a 7–30 power stereoscope. We sorted diet components into nine categories; 1) rodents, 2) ungulates, 3) invertebrates, 4) graminoids, 5) forbs, 6) shrubs, 7) roots, 8) seeds, and 9) crops. Other infrequent items like fish and garbage were noted separately. Where possible we differentiated rodents into golden marmots and others. We estimated the percent relative volume (RV) of these diet categories visually, which is known to correspond well to actual volumes [45]. We calculated the Relative Frequency (RF) of each diet component as the total number of occurrences divided by the total scat samples.

Genetic analysis (the *trn*L approach): The 63 fecal samples were used in this study, which were previously typed by microsatellites [36]. Total DNA was extracted from about 10 mg of a feces sample with the DNeasy Tissue Kit (Qiagen GmbH, Hilden, Germany), following the manufacturer's instructions. The DNA extracts were recovered in a total volume of 300 μL. Mock extractions without samples were systematically performed to monitor possible contaminations. DNA amplifications were carried out in a final volume of 25 μl, using 2.5 μl of DNA extract as a template. The amplification mixture contained 1 U of AmpliTaq® Gold DNA Polymerase (Applied Biosystems, Foster City, CA), 10 mM Tris-HCl, 50 mM KCl, 2 mM of MgCl_2_, 0.2 mM of each dNTPs, 0.1 μM of each primer, and 0.005 mg of bovine serum albumin (BSA, Roche Diagnostic, Basel, Switzerland). The mixture was denatured at 95°C for 10 min, followed by 35 cycles of 30 s at 95°C,and 30 s at 55°C; the elongation was removed in order to reduce the +A artefact [46,47]. Each sample was amplified with primers *g* and *h* [38], modified by the addition of a specific tag on the 5' end in order to allow the recognition of the sequences after the pyrosequencing, where all the PCR products from the different samples are mixed together. These tags were composed of six nucleotides, always starting with CC on the 5' end, followed by four variable nucleotides that were specific to each sample.

PCR products were purified using the MinElute PCR purification kit (Qiagen GmbH, Hilden, Germany). DNA quantification was carried out using the NanoDrop® ND-1000 UV-Vis Spectrophotometer (NanoDrop Technologies® Wilmington, DE). Then, a mix was made taking into account these DNA concentrations in order to obtain roughly the same number of molecules per PCR product corresponding to the different feces samples.

Large-scale pyrosequencing was carried out on the 454 sequencing system (Roche, Basel, Switzerland) following manufacturer's instructions, and using the GS 20. From the mix of sequences obtained after the pyrosequencing, the first step in the data analysis consisted of dispatching the different sequences according to the tag present on the 5' end of the primers. Thus, for each sample (each feces), a file was generated, containing all the sequences having the relevant tag on its 5' end. Then, these sequences were analyzed to determine the diet. Only sequences present more than three times were taken into account in the subsequent analyses. To determine bear diet, the sequences were first compared to the reference database and then, if no match was found, to public databases, using the MEGABLAST algorithm [48].

We plotted the frequencies of identified families and classified them as regular (≥10% occurrence) and occasional diet items (<10% occurrence) for brown bears. Families with >50% frequency were considered as preferred plant food for bears. Bellemain et al. found a significant negative correlation between the freshness of fecal samples and the proportion of positive amplification as well as between the freshness of fecal samples and the quality index [36]. In this study we tested whether the number of plant species identified from a sample were related to the freshness of the sample.

### Energy contribution to the diet

Diet items differ greatly in their digestibility [6,49] and nutritional composition [50], which biases scat analysis. To adjust for differential digestibility of diet items, we estimated the Dietary Content (EDC) by applying Correction Factors (CF) proposed by [49] to RV. We used the following CFs: 4 for rodents, 3 for ungulates, 1.1 for invertebrates, 0.24 for graminoids and crops, 0.26 for forbs, 1 for roots, and 1.5 for seeds.

We estimated the energy contribution of each component of diet, by multiplying the EDCs by their respective estimated digestible energy values. For animal matter we used digestible energy values reported in [50]; ungulates = 29.4 kj/g, rodents = 22.1, and invertebrates = 17.7 [51]. The digestible energy (kj/g) for plants in DNP was estimated as; graminoids:11.8, forbs: 11.2, and shrubs: 12.2 [18].

### Sex variation

Bellemain et al. [35] identified 28 individual bears from DNA in fecal samples. Because we used the same samples in the present study, we could investigate sex differences in diet. We ran a table analysis (PROC FREQ) in SAS (SAS Institute Inc.) and computed Fisher’s exact text and odds ratios between sexes [52]. Fisher’s exact test was chosen due to small sample size for individual diet categories.

### Temporal variation

We grouped the data into four months (June through September) to investigate whether there was a temporal trend in diet selection. We had few samples for October, which we included in September. We tested only five categories (rodents, graminoids, forbs, roots, seeds) with > 10% overall frequency. Although food was a multicategory response, diet categories are not mutually exclusive in one sample. Therefore we could not use a multicategory logit model [52]. We treated each category as a binary response and ran five logistic models for each diet category. Letting *π* denote probablity of finding a diet component, we tested temporal impact using equation 5.4.3 in Agresti [52]:

$$Logit\;(\pi )=\alpha +\beta_{i}^{x};\;x=\mathrm{factor}\;\mathrm{of}\;\mathrm{month}\;\mathrm{with}\;\mathrm{levels}\;i=1,2,3,4\;(\mathrm{June}‐\mathrm{September})$$

We ran PROC GENMOD procedure in SAS to estimate the parameters. The probability of finding a particular diet component in each month (${\hat{\pi }}_{i}$) was calculated as:

$${\hat{\pi }}_{i}=\frac{e^{\alpha +\beta_{i}^{x}}}{{1+e}^{\alpha +\beta_{i}^{x}}}$$

The samples used in the *trn*L approach were collected only between July to September. We counted the number of species and families in each group and compared them across the months.

### Habitat variation

Deosai is represented by four vegetation classes (marshy, grassy, stony, rocky), and each class has its own characteristic floral species composition and cover [35]. We plotted the locations of fecal samples on a vegetation map in Arc GIS (ESRI Inc., 2006) to determine the habitat type they were found in (marshy, grassy, stony, rocky, and valley). Habitat differences in diet contents were investigated using logistic regressions, following the same procedure as described for the temporal variation.

## Results

### Mammalian biomass

A small population of Himalayan ibex was present in the hills east of DNP and in the surrounding valleys. We recorded 12 sightings and 20 signs (including one dead ibex) within the park in 1999–2005 and 4 sightings in the surrounding valleys in 2005. We estimated about 25–30 individuals within the park and 50–70 in the surrounding valleys of Bubind, Minimerg, and Karabosh. Musk deer prefer forests, so they were not present in the park. We counted 18 deer in 12 sightings on 7 transects in the surrounding valleys in 2005, where we estimated a population of 20–30. Thus, the biomass of wild ungulates in and around DNP was approximately 8 tons (1.4 kg per km^2^ within the park area).

Based on 33 transects (271 km length) we conducted during 2004–2006, we estimated golden marmot density at 79.7±4.6 individuals per km^2^. This density corresponds to a biomass of 250 kg/ km^2^. The rocky habitat was generally devoid of marmot colonies, density was similar in grassy and stony habitats (20 and 18 colonies per km^2^, respectively) but the highest in marshy habitat (26 colonies per km^2^). The three habitats supporting marmots cover about 65% of the park [35]. Multiplying the biomass estimate by the total productive area resulted in an estimate of 250 tons of marmot biomass for the entire park (about 300 kg /km^2^), which is about 60 times higher than the biomass of the largest mammal (brown bear).We recorded sign of brown bear digging at 33% of the colonies, a density of 6.7 dug colonies per km^2^.

### Diet composition and energy contribution

We analyzed a total of 334 brown bear scats collected over four years (101, 114, 49, and 70 in 2003–2005 and 2007, respectively). The average scat volume was 139 ml (SD: 52). Most of the scats (70%) were composed of only plant residues. Graminoids (grasses and sedges) had the highest frequency (93%) and constituted the bulk (85%) of the scat residues (Table 1) and forbs had the second highest frequency, 52% (presence recorded by stems and inflorescence only). The volume of animal residues was only 4%, with rodents constituting most (88%) of it. About 30% of the rodent residues were those of golden marmots, and rest could not be identified. We found remains of fish in 2 scats, birds in 3, and 4 scats contained garbage (plastics and food packing).

**Table 1:** Relative frequency (RF), relative volume (RV) and estimated dietary content (EDC) of diet items in brown bear scats from Deosai National Park, Pakistan.

		RF (%)	RV (%)	EDC (%)
Animal Matter		26.6	4.1	36.5
	Rodents	19.2	3.4	32.5
	Ungulates	6.9	0.5	3.9
	Invertebrates	6.9	0.1	0.2
Plant Matter		100.0	95.9	63.5
	Graminoids	92.8	85.3	48.5
	Forbs	51.5	0.9	0.6
	Shrubs	3.9	0.0	0.0
	Roots	20.1	4.3	10.2
	Seeds	24.6	0.4	1.3
	Crops	5.7	5.0	2.9

We could not differentiate plant matter taxonomically by scat analysis beyond the general categories of graminoids and forbs. However, with the *trn*L approach, we found a total of 57 plant taxa in the bear feces, belonging to 50 genera and 29 families (Table 2). The *trn*L approach allowed us to identify 47% of the plants to species level, 74% to genera, 77% to tribe, 82% to subfamily, and all to family (Table 2). Thirty-one species sequences were identified from the reference database of plants from the DNP and the remaining 26 species were the closest matches from public databases.

**Table 2:** A complete list of plant species identified by the *trn*L approach in the diet of brown bears in Deosai National Park, Pakistan.

Family	Species	Rank^1^	Frequency	Food Type	Identification source^2^	Comments
Actinidiaceae	Actinidia	Genus	0.02	Fruit	Public database	*Actinidia chinensis* and *Actinidia deliciosa* both species found in Pakistan
Adoxaceae	Adoxaceae	Family	0.02	Forb	Public database	Not recorded from Pakistan yet, but one taxon *Adoxa moschatellina* is expected to occur.^a^
Apiaceae	Apioideae	Subfamily	0.05	Forb	Reference	Also known as Umbelliferae, represented in DNP by 18 species.^b^
Apiaceae	Heracleum candicans	Species	0.50	Forb	Reference	
Araliaceae	Araliaceae	Family	0.02	Forb	Public database	Not recorded from DNP, but three taxa (Aralia cachemirica, Hedera nepalensis, Schefflera bengalensis) are expected to occur in the area.^a^
Asteraceae	Leontopodium brachyactis	Species	0.02	Forb	Reference	This family is represented by 93 species in DNP, including this one.^b^
Asteraceae	Asteraceae	Family	0.23	Forb	Public database	
Brassicaceae	Thlaspi andersonii	Species	0.02	Forb	Reference	This species has been documented from DNP, along with other six species from this family.^b^
Caryophyllaceae	Cerastium cerastoides	Species	0.13	Forb	Reference	
Caryophyllaceae	Cerastium pusillum	Species	0.10	Forb	Reference	
Caryophyllaceae	Cerastium sp.	Genus	0.05	Forb	Reference	
Crassulaceae	Rhodiola sp.	Genus	0.02	Forb	Public database	
Cupressaceae	Cupressaceae	Family	0.03	Other	Public database	Three juniper species (*Juniperus communis*, *J*. *Excelsa*, *J*. *Turkestanica*) are documented from DNP.^b^
Cyperaceae	Carex diluta	Species	0.63	Graminoid	Reference	31 species of Cyperaceae, including Carex diluta, are documented from DNP.^b^
Cyperaceae	Carex	Genus	0.61	Graminoid	Public database	
Ephedraceae	Ephedra gerardiana	Species	0.02	Browse	Reference	Two species (*Ephedra gerardiana*, *E*. *Intermedia*) are present in DNP. Possible source for berries.^b^
Euphorbiaceae	Euphorbia sp.	Genus	0.02	Forb	Public database	Four species (*Ephorbia comigera*, *E*. *kanaorica*, *E*. *thomsonianum*, *E*. *Tibetica*) are documented in DNP.^b^
Euphorbiaceae	Euphorbiaceae	Family	0.02	Forb	Public database	
Fabaceae	Astragalus rhizanthus	Species	0.05	Forb	Reference	Also known as Papilionaceae.
Fabaceae	Oxytropis cachemiriana	Species	0.02	Forb	Reference	
Fabaceae	Galegeae	Tribe	0.03	Forb	Public database	
Fabaceae	Glycine sp.	Genus	0.02	Forb	Public database	
Griseliniaceae	Polysoma sp.	Family	0.03	Browse	Public database	
Juncaceae	Juncus sp.	Genus	0.02	Graminoid	Public database	Three species (Juncus articulatus, J. membranaceus, J. Sphacelatus) are recoded in DNP.^b^
Labiatae	Mentheae	Tribe	0.15	Forb	Reference	Either of Nepeta linearis or Thymus linearis are possible, because both have same molecular sequence.
Lycopodiaceae	Lycopodiaceae	Family	0.02	Other	Public database	Moss
Orobanchaceae	Pedicularis albida	Species	0.02	Forb	Reference	Scrophulariaceae
Orobanchaceae	Pedicularis sp.	Genus	0.02	Forb	Public database	
Papaveraceae	Papaver nudicaule	Species	0.02	Forb	Reference	
Pinaceae	Cedrus sp.	Genus	0.03	Tree	Public database	*Cedrus deodar* is the only species in this genus, found in the surrounding valleys of DNP.^a^
Plantaginaceae	Plantaginaceae	Family	0.02	Forb	Public database	
Poaceae	Agrostis vinealis	Species	0.31	Graminoid	Reference	Poaceae is represented by 42 species in DNP.^b^
Poaceae	Elymus longi-aristatus	Species	0.23	Graminoid	Reference	Elymus longi-aristatus and Triticum (wheat) have same sequence, so wheat crop could be another possibility.
Poaceae	Koeleria macrantha	Species	0.05	Graminoid	Reference	
Poaceae	Poa alpina	Species	0.02	Graminoid	Reference	
Poaceae	Poa supina	Species	0.47	Graminoid	Reference	
Poaceae	Pooideae	Sunfamily	0.92	Graminoid	Public database	
Poaceae	Poa sp.	Genus	0.02	Graminoid	Public database	
Poaceae	Poa sp_91E	Genus	0.02	Graminoid	Public database	
Poaceae	Stipeae	Tribe	0.03	Graminoid	Public database	
Polygonaceae	Aconogonon rumicifolium	Species	0.23	Forb	Reference	23 species of Polygonaceae are present in DNP.^b^
Polygonaceae	Bistorta affinis	Species	0.47	Forb	Reference	
Polygonaceae	Polygonum cognatum	Species	0.03	Forb	Reference	
Polygonaceae	Rumex nepalensis	Species	0.18	Forb	Reference	
Polygonaceae	Polygonaceae	Species	0.34	Forb	Public database	
Ranunculaceae	Aconitum violaceum	Species	0.05	Forb	Reference	
Ranunculaceae	Thalictrum sp.	Genus	0.02	Forb	Public database	Two species (*Thalictrum alpinum*, *T*. *foetidum*) are documented in DNP.^b^
Rosaceae	Alchemilla sp_67E	Genus	0.02	Forb	Reference	
Rosaceae	Cotoneaster affinis	Species	0.02	Forb	Reference	
Rosaceae	Rosoideae	Sunfamily	0.05	Forb	Public database	
Rubiaceae	Galium boreale	Species	0.10	Forb	Reference	
Rubiaceae	Galium sp.	Genus	0.03	Forb	Reference	
Rubiaceae	Rubiaceae	Family	0.02	Forb	Public database	
Rutaceae	Rutaceae	Family	0.02	Other	Public database	Cultivated (citrus, etc)
Salicaceae	Salix sp.	Genus	0.02	Browse	Reference	
Saxifragaceae	Saxifraga flagellaris	Species	0.02	Forb	Reference	Represented by seven species in DNP.^b^
Saxifragaceae	Saxifraga hirculus	Species	0.06	Forb	Reference	

^1.^ Level to which plant was identified

^2.^ Source of identification for DNA sequences; Reference (database of 91 plants from DNP), Public databases for finding closest match (Zhang et al. 2000).

^a.^ Flora of Pakistan (http://www.efloras.org/flora_page.aspx?flora_id=5)

^b.^ [37].

**Fig 1:**
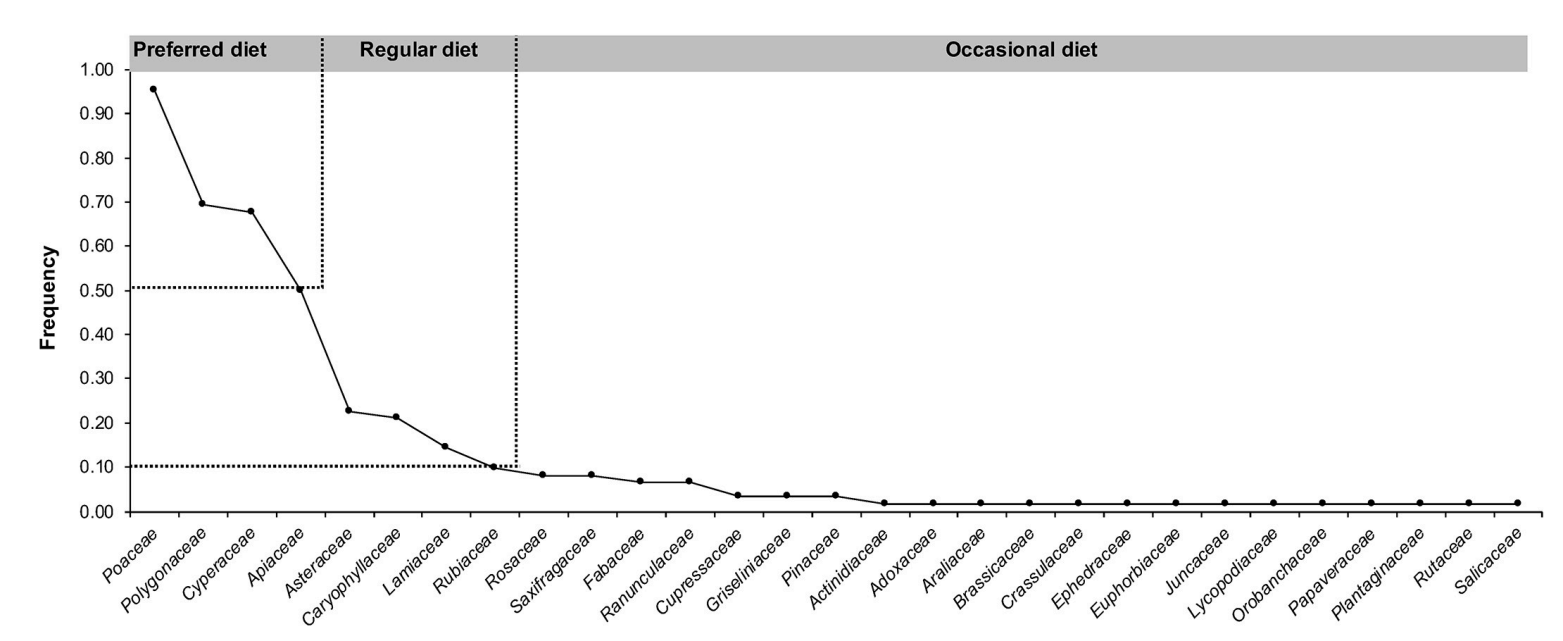
A frequency plot of plant families in the diet of brown bears in Deosai National Park, Pakistan, identified by the *trn*L approach.

The 57 plant species were not evenly represented in the diet; the frequencies ranged from 2–92%. About 70% of the identified species were represented by ≤ 3 samples, and among them 27 species were represented by single samples. There were only four species with occurrence in more than 50% samples; one unidentified species of Poaceae, two species of Cyperaceae (*Carex diluta*, *Carex* sp.), and one species of Apiaceae (*Heracleum candicans*). The unidentifed grass (subfamily Poideae) had the highest frequency (92%). The dietary diversity at the generic level was similar; *Carex*, *Heracleum*, and one Poaceae genus (unidentified) were the only genera represented in more than 50% of the samples. Among the 29 identified families, 14 were represented by only one sample. The regular plant diet (≥ 10% occurrence) of brown bears was comprised of only 8 families; Poaceae, Polygonaceae, Cyperaceae, Apiaceae, Asteraceae, Caryophyllaceae, Lamiaceae, and Rubiaceae (Fig 1). The first four families constituted the preferred diet, with more than 50% occurrence. We did not find any correlation between age of the sample (fresh, 2–3 days old, 1-week old) and number of plant species identified (Spearman r = -0.5, P = 0.66).

The relative contribution to the energy assimilation was almost equal for animal (54%) and plant (46%) components of the diet. Rodents (48%) and graminoids (33%) were the main sources of energy for bears. Ungulates (7.7%) and roots (7%) were second, and other components were not important. The energy gained by brown bears per gram of ingested food was estimated at 14.8 kj.

### Sex differences in diet

**Fig 2:**
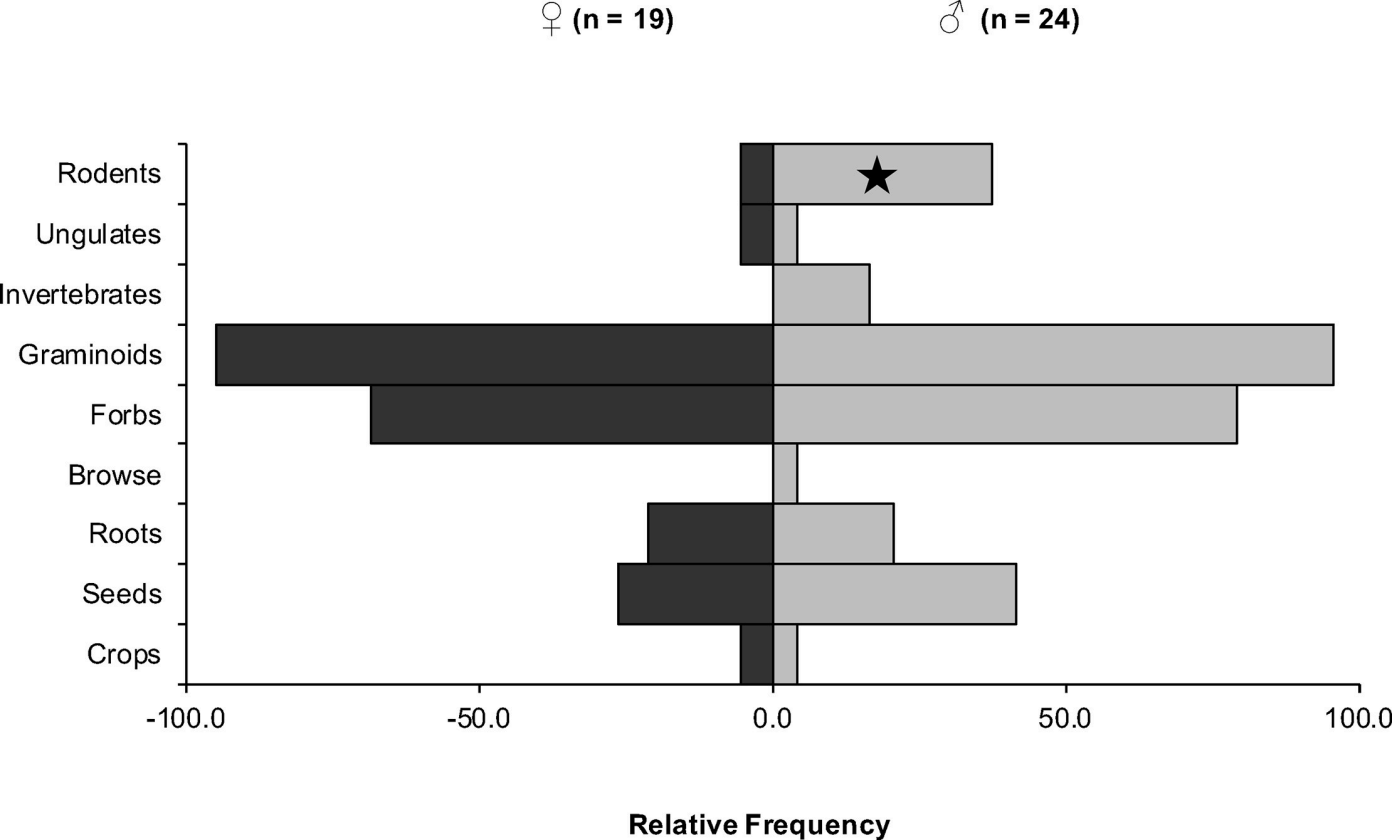
Sex differences in the diet of brown bears in Deosai National Park, Pakistan, based on scat analysis. Star indicates significant difference.

Scat analysis of 43 samples, for which sex was known (Fig 2), indicated that the foraging behavior of the sexes was quite similar, except for rodents (P = 0.02, the Fishers’s exact test). Females’ likelihood of eating rodents was 84% lower than that of males (Odds ratio: 0.16).

Among the 62 fecal samples analyzed by the *trn*L approach, 21 belonged to females, 37 to males, and for 4 sex was not known. We identified 34 and 43 species from female and male samples, respectively. The ratio of graminoids to forbs did not differ significantly (χ^2^: 0.24, P = 0.63) among sexes. Comparing individual species, the Fisher’s exact test indicated significant differences in three plant species. The likelihood of eating *Bistorta affinis* (Odds ratio = 0.30, P = 0.02), *Carex diluta* (Odds ratio: 0.34, P = 0.03), and *Carex* sp. (Odds ratio: 0.24, P = 0.01) was significantly higher for males.

### Temporal variation

**Fig 3:**
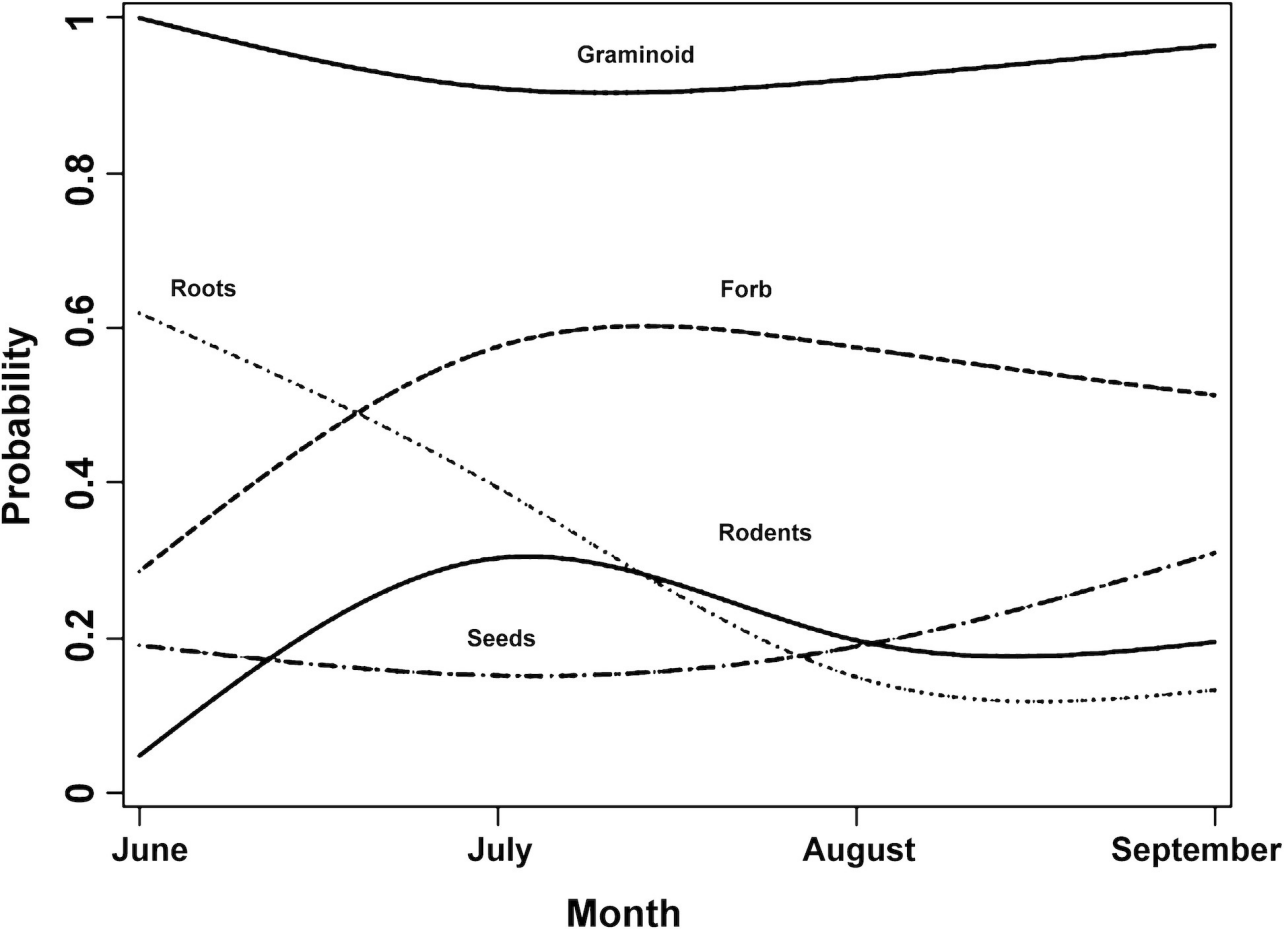
Temporal trend in probabilities of major diet categories of brown bears in Deosai National Park, Pakistan, based on scat analysis.

The predicted probabilities of diet items depicted a divergent pattern (Fig 3). In the beginning of the season, the diet was dominated by graminoids and roots, and became more diverse in July. The frequency of roots was 10 times higher in June compared to September (exp (2.3624), Table 3). However, the logistic regressions indicated a lack of significant temporal effect on major diet components, except for roots, which showed a decline in occurrence late in the season (Table 3).

**Table 3:** Parameter estimates of logistic regression models of the temporal effect on major diet categories of brown bears in Deosai National Park, Pakistan. September was set as the base redundant parameter.

Parameter	Rodents	Graminoid	Forb	Roots	Seeds
Intercept	-1.4198*	3.3051*	0.0531	-1.8769*	-0.8014*
June	-1.5759	23.0603	-0.9694	2.3624*	-0.6456
July	0.5869	-1.0025	0.2523	1.4461*	-0.9214
August	0.0137	-0.8455	0.2484	0.1392	-0.6553
Model Fit	Good	Good	Poor	Good	Good
G^2^	285.92	124.71	399.89	267.85	311.51
P-value	0.56	1.00	0.00*	0.820	0.184

*P-value < 0.05

Also, in the *trn*L data, we did not find a temporal difference in the number of plant species (χ^2^: 2.54, P = 0.77) or families (χ^2^: 2.2, P = 0.82). However, the ratio of graminoid forage to forbs changed significantly over three months (Spearman's r: -0.82, P = 0.04), favoring forbs later in the season. Four families showed a temporal trend; Asteraceae (r: -0.522) and Poaceae (r: -0.309) declined late in the season, whereas Polygonaceae (r: 0.714) and Fabaceae (r: 0.617) showed an increasing trend. The higher frequency of the latter two families might account for a higher frequency of seeds in the scats late in the season.

### Habitat variation

The four habitats in DNP were homogenous with respect to diet contents of scats (Wald Statistics ranged 0.14–2.94 with P-values 0.15–0.70, for all parameters tested in logistic regressions). However, the diet in valleys (n = 43) was significantly different from DNP (n = 188). In the surrounding valleys, we found higher likelihood of eating graminoids (β = 2.0471, Wald Statistics = 30.83, P <0.01), and lower likelihoods for rodents (β = -3.127, Wald Statistics = 38.39, P <0.01), roots (β = -2.0305, Wald Statistics = 30.31, P <0.01), and seeds (β = -2.4563, Wald Statistics = 35.64, P <0.01). The frequency of forbs did not differ (Wald Statistics = 0.344, P = 0.55). Thus, DNP provided more nutritious and diverse food to bears than the surrounding valleys.

Of the 62 fecal samples used in the *trn*L approach, 15, 16, 13, and 7 were collected from marshy, grassy, stony, and rocky habitats within the park, respectively. Ten were from surrounding valleys and location of 1 sample was not recorded. Neither the number of species (χ^2^:1.52, P = 0.82) nor the number of families (χ^2^:1.85, P = 0.76) varied significantly across habitat types. However, four families, Adoxaceae, Araliaceae, Ephedraceae and Orobanchaceae, were represented by single samples and were present only in the valleys. Pinaceae and Cupressaceae also occur only in valleys, although the fecal samples were collected from the park. The ratio of graminoids to forbs in the diet did not vary significantly (χ^2^:1.35, P = 0.72) among the four habitats of the park, however samples from the surrounding valleys showed a significantly higher proportion of graminoids (χ^2^:24.4, P <0.01).

### Comparing the classical scat analysis and the trnL approach

Forty-three scat samples, analyzed by both techniques, provided an opportunity to compare classical scat analysis and *trn*L approach. The frequencies of graminoids, forbs and shrubs obtained by the *trn*L approach were 98, 84 and 7%, respectively, compared with 93, 61 and 5%, respectively for the scat analysis. In the scat analysis, three samples lacked graminoids. Two of these samples were composed solely of crop residues and one was dominated by animal remains. Brown bears used three crops from the valleys surrounding DNP; wheat (*Triticum aestivum)*, corn (*Zea mays*), and barley (*Hordeum vulgare*), all of which belong to the Poaceae family. By adding these two crop samples to “graminoids” in the scat analysis data, the frequency of graminoids became identical in both methods.

There was a large difference in frequencies of forbs determined by the two methods. In the scat analysis, the frequency of forbs was dependent upon the identification of herbaceous plants based only on the occurrence of stems or inflorescences. Two other categories of diet; seeds and roots, likely also belonged to forbs. When we pooled these three categories, the frequency rose to 75%, but remained lower than the *trn*L frequency (84%). We conclude that the *trn*L approach verifies the findings of the scat analysis concerning graminoids and shrubs, but the scat analysis underestimated the occurrence of forbs due to relatively low volumes in the scat (about 1%). Both methods agreed that the occurrence of forbs increased in the late season, and graminoids occurred at higher frequencies in the valleys.

## Discussion

### Diet composition

The *trn*L approach and classical scat analysis are complementary techniques, and together can provide a comprehensive understanding of feeding ecology of an omnivorous species such as brown bear. The *trn*L approach provided a more accurate descrption of plant diversity in the diet and its frequency. The scat analysis helped ascertain relative volumes of major diet groups, particularly the animal prey, which could not be determined by the *trn*L approach.

The brown bear diet was quite diverse in DNP, represented by 57 plant species, insects, ungulates and several rodent species. Poaceae, Polygonaceae, Cyperaceae, and Apiaceae are the commonly eaten families. However, the adjusted diet content indicated that only graminoids (represented by sedges and grasses) and golden marmots comprised the bulk of the diet. Golden marmots, though relatively low in frequency, had the highest contribution to digestible energy.

Food selection in animals is a function of availability, handling time, and quality [53,54]. However, in the case of omnivores, availability is the key factor in diet selection, because their food varies between relatively rare but high-quality animal matter and abundant low-quality vegetation [5]. Looking at plant and animal resources separately, we found consumption in accordance with availability. Graminoids comprised the highest biomass in park, followed by forbs [35]. Shrubs, which are restricted to narrow stream belts, are poorly represented in diet. Fruit plants are also not available in the park. There were three plants in the diet that could be a source of fruits for bears; *Ephedra gerardiana*, *Actinidia* sp., and an unidentifed species of Griseliniaceae, but these were represented by few samples (frequency <0.03). When Deosai National Park was established in 1993, there was no resident population of ungulates [55]. A small population of ibex was occasionally visiting, which has recently increased to 25–30 individuals and inhabits the eastern hills of the park. Therefore, there was no substantial and predictable ungulate prey available to the bears in the park. Domestic livestock were guarded by dogs and shepherds in DNP. The golden marmot represented the major biomass of available mammals and comprised the main component of animal matter in the diet. The DNP has a great variety of invertebrate fauna, the abundance of different groups changes seasonally, but a continuous supply is available [41]. They did not make a substantial part of the bear diet, probably because they did not occur in an aggregated form, like anthills in Sweden [56] or moth aggregation sites in North America [57], where they make a significant contribution to energy assimilation in brown bears.

The *trn*L approach indicated that scat analysis underestimated the occurrence of forbs in the diet of brown bears, at least by 10%. Likewise, we might have underestimated their volume in the scats, which is a limitation of scat analysis that has been reported earlier [58]. However, underestimation of the volume of forbs may not have been substantial, because the following observations support the conclusion of the scat analysis that graminoids comprised the bulk of the food. First, habitat use usually is determined by the distribution of the main food plants [59,60], though those plants might be eaten due to their greater availability rather than selective preference [12]. We documented that brown bears prefer marshy habitats in DNP [35]. The marshy habitat, with predominantly graminoid vegetation, has the highest biomass production in DNP (3919 kg dry matter/km^2^). It covers only 15% of the park but produces half of its vegetation biomass [35]. Secondly, during a time budget study, bears were mostly observed in marshy habitats where their dominant activity was grazing [43]. Thirdly, the highest density of brown bears occurs in the Black Hole area (central part of the park), which is predominantly a marshy habitat [18]. Thus, the graminoids are the most abundant and concentrated source of food for bears, and apparently the key factor explaining resource selection by brown bears in DNP. In agreement with our results, brown bears using alpine habitat in Alaska are heavily dependent on graminoids [61].

Vertebrates that depend on plant matter for their nutritional requirements exhibit digestive track modifications, either through compartmentalization of the for-e gut or an elaborate sacculation of the hind-gut [62]. These specializations aid in the retention of digesta and harbor microbial populations that convert indigestible plant matter (cell wall components) into absorbable nutrients [62,63]. The brown bear possesses an anatomically simpler gastrointentinal tract like other carnivores [62,64]. Although two adaptations, an extremely large intestine and bunodont molars, allow the brown bear to utilize plant matter more efficiently than other carnivores, it has a limited capacity for microbial digestion. To overcome the limitation of low digestibility, herbivores like perissodactyles and omnivores (raccoon *Procyon lotor*, pig *Sus scrofa*, etc) respond by increasing consumption [63,65]. This strategy sacrifices retention time but enables animals to utilize the cell contents. The most extreme adaptation to high intake (up to 6% of body weight) and low extraction (8±3 hours of retention) has been observed in the giant panda *Ailuropoda melanoleuca* [63,66]. The retention time of plant food in brown bears is also very short (7±0.8 hour, 48), however the relatively larger intestine may increase absorption [63,67]. The retention time in brown bear is 72% and 86% shorter than in horses and ruminants, respectively (retention times in horse and sheep/goat are 25 and 50 hours, respectively, [62,67,68] Although the digestion of structural carbohydrates is insignificant in brown bears due to fast passage [47], the loss of cell soluble is small (protein digestion is only 5% lower than in ruminants, [48]. The high intake rate of brown bears is supported by a time budget study in DNP [43], where bears were observed spending the largest part of the day foraging (67% of daylight hours) and foraging was predominantly grazing (96.3%). Brown bears therefore would require a consistent source of large amount of vegetation, which is provided by the marshy habitats in DNP.

Brown bears are sexually dimorphic [9], males are about 50% heavier than females in DNP [18]. Larger body size increases the reproductive success of males through; 1) increasing chances of fertilization in a promiscuous mating system [10,69,70]because ejaculate volume is correlated with size [71], and 2) increasing social dominance, which increases access to reproductive females [10]. The more carnivorous food of males was probably an effect of their larger body size. Maintaining larger body size requires more energy, which is met by meat [19]. Golden marmots made up the major meat source in DNP and capturing requires much soil digging (soil heaps up to 1 m height can be observed in a marmot colony). Large and stronger bears might be more efficient in digging marmot colonies.

Seasonal variation in diet composition has been reported for brown bears in areas where the seasonal abundance of food changes considerably or bears shift their habitat seasonally [5,10,72]For example, in central Sweden; ungulates comprise the main diet in spring, whereas ants, forbs, and ungulates dominate in summer, and berries dominate the autumn diet [8]. In DNP, we did not find a significant temporal influence, probably because the availability of major food item (graminoids) did not change over the months. Graminoids in moist places (like marshy habitats in DNP) remain physiologically active, thus higher in protein content, even during post-growing season [4,73]. During the late growing season, before denning, bears show hyperphagia [74] and may increase their intake of high nutritious food (meat) if available [5]. Therefore, we expected higher consumption of meat (marmots) during the later months. Golden marmots are very sensitive to low body temperature and hibernate socially in a single hibernaculum [42], which prevents body temperatures falling below a critical threshold through coordinated bouts of social thermoregulation [75,76]. Blumstein and Arnold [42]reported, from an area close to DNP, that above-ground activity of marmots becomes limited by the first week of September, and they start plugging burrows for hibernation by the second week of September. Although brown bears foraged until October in DNP, the limited activity by marmots probably explains the lack of increase in meat intake in later months.

Brown bears eat anthropogenic foods where they coexist with humans [9]. Human-related food in the present study was predominantly crops, and in a few scats, we found cultivated fruits (citrus, kiwi) and garbage (food packaging). Residues of ungulates in scat, may also belong to domestic livestock. Brown bears usually do not attack livestock in our study area, because livestock are guarded by shepherds and dogs. They might therefore have scavenged livestock carcasses. Brown bears steal yoghurt, which people keep in open bags of goat/sheep skins for drying, from villages and shepherd huts. The DNP has neither settlements nor agriculture within the park area. All communities and their cultivations are in surrounding six valleys [18]. The majority of brown bear dens are also present in those valleys. Thus, brown bears stay in the valleys in early spring, after denning, and they raid crops at that time.

In conclusion, the brown bear diet in DNP is predominantly based on carbohydrates, and the protein content was low compared with other brown bear populations with comparable data (Table 4). However, Westerterp and Kayser [77] suggested that carbohydrates are a better energy source than proteins at high altitudes, because of their low thermogenesis values (5–10% for carbohydrates, and 20–30% for proteins), and because they require less oxygen to metabolize, which is an advantage in the low-oxygen environment of high altitudes. A carbohydrate-rich diet increases the respiratory quotient, which thus provides high oxygen saturation in the blood [78].

**Table 4:** Comparison of energy assimilation in the brown bear population of Deosai National Park with other brown bear populations from Asia, Europe and North America. Energy assimilated per gram of ingested food was calculated for these studies by applying correction factors [49] and energy estimates of food items [51] to relative percent volumes.

Study Area	Diet Composition (%Volume)			Energy*	Rep. Rate**	Reference
	Veg	Fruit	Animal	(kj/g)		
**Asia**						
Deosai National Park, Pakistan	95.9	-	4.1^d^^,^^e^^,^^f^	14.8	0.23	Present study; [39]
Kekexili Nature Reserve, China	2	-	98 ^d^^,^^e^	25.6		[14]
Chang Tang Reserve, China	26.2	-	73.8 ^d^^,^^e^	22.8		[82]
Southern Hokkaido, Japan	72.3	17 ^a^	10.7 ^c^^,^^e^^,^^f^	20.9		[12]
Northern Hokkaido, Japan	48.3	46.2 ^a^	5.5 ^c^^,^^d^^,^^e^	19.3		[13]
Western Tian Shan, Central Asia	22	55.7 ^a^	20.8 ^d^^,^^e^^,^^f^	21.1		[85]
Northern Tian Shan, Central Asia	60.9	20.5 ^a^^,^^b^	18.6 ^e^	20.6		[85]
Caucasian Reserve, Russia	35	53 ^a^^,^^b^	12 ^e^	23.9		[85]
Eastern Sayans, Russia	28.9	38.7 ^a^^,^^b^	32.4 ^e^^,^^f^	23.5		[85]
Western Sayans, Russia	34.4	54.8 ^a^	10.8 ^d^^,^^e^^,^^f^	24.3		[85]
Far East, Russia	23.5	43.2 ^a^^,^^b^	33.4 ^e^^,^^f^	25.5		[85]
**Europe**						
Central Sweden	43.6	26.7 ^a^	29.7 ^e^^,^^f^	20.1	0.96	[8,86]
North-eastern Norway	20.9	38.1 ^a^	41 ^e^^,^^f^	25.1		[87]
Nord-Trøndelag, Norway	33.3	16 ^a^	50.7 ^e^^,^^f^	26.6		[8]
Central-south Norway***	25	39 ^a^	36 ^d^	20.5		[88]
Riaño National Hunting Reserve, Spain	45.5	40.6 ^a^^,^^b^	13.9 ^e^^,^^f^	24.4		[7]
Cantabrian Mountains, Spain	34.1	56 ^a^^,^^b^	9.9 ^e^^,^^f^	24.0		[89]
Yugoslavia (Croatia)	29.1	68.7 ^a^^,^^b^	2.2 ^e^^,^^f^	22.8		[58]
**North America**						
Northern Yukon, Canada	76.4	20.3 ^a^	3.3 ^d^^,^^e^^,^^f^	16.9	0.50	[24,25,90]
West-central, Alberta, Canada	65.5	21.9 ^a^	12.7 ^e^^,^^f^	21.3		[91]
Banff National Park, Canada	65	25 ^a^	10 ^e^	21.3	0.48	[4,31]
Flathead River Drainage, BC, Canada	52	29 ^a^	19 ^d^^,^^e^^,^^f^	22.6	0.85	[5,92]
Yellowstone 1973–74, USA	80.1	6.1 ^a^^,^^b^	13.8 ^c^^,^^d^^,^^e^	22.7	0.62	[6,93]

* average energy per gram of ingested food

**number of cubs/female/year

***Adjusted volume after applying correction factors

^a^soft mast

^b^hard mast

^c^fish

^d^rodents

^e^ungulates

^f^invertebrates

### Energy assimilation and life history

The positive role of meat in the reproductive performance of female brown bears is well documented [17,19,79]. Mast is the second most important source of protein and energy and is consumed by brown bears in most populations in large quantities. For example, berries make 82% of the autumn diet in central Sweden [8], and pine nuts make up to 45% scat volume in Yellowstone [80]. Brown bears with access to abundant salmon are also reported to feed extensively on fruits (87% fecal volume, [81]). Robbins et al. [15] documented that mixed diets (salmon and fruits) contribute to 72% higher growth in brown bears as compared to a meat-based diet, and this effect is most pronounced in small-sized bears. A comparison of six brown bear populations (Table 4), indicated that the reproductive rate was positively related to the amount of animal matter (r = 0.86), fruits (r = 0.74) and digestible energy (r = 0.66) in the diet, and negatively related to the amount of vegetation in the diet (r = -0.910).

The energy in the diet of 22 brown bear populations ranged between 16.9–26.6 kj/g (average = 22.5) (Table 4). The predominantly carnivorous populations, like two populations of the Tibetan Plateau [14,82], have higher levels of digested energy. The brown bear population in DNP, which lacks fruits in its diet and has relatively little meat, assimilates the lowest amount of energy per unit ingested food of all brown bear populations with comparable data (Table 4). High-altitude populations, with a low nutritious diet and facing extreme environmental conditions, are expected to have poor reproductive performance [17,24]. These factors probably contribute to the very low reproductive rates of the brown bear population in DNP [18].

The Central Asian populations, which are closer to the Himalayan brown bear genetically and geographically [16,83], have access to mast and consequently higher levels of food energy (Table 4). Thus, the poor nutrition of Himalayan brown bear in DNP cannot be generalized for its entire range. Brown bears in forested areas of Himalaya might have better nutrition than in DNP, because these areas have wild ungulates and a variety of fruit-bearing plants. For example, Schaller [84] reported frequencies of markhor (*Capra falconeri*) and ibex at 17% and 16%, respectively, in scats of brown bear from Chitral Gol and Baltoro (both locations in Pakistan). However, he concluded that graminoids comprised the bulk of brown bear diet there.
