# Correction: Diet of the brown bear in Himalaya: Combining classical and molecular genetic techniques

**DOI:** 10.1371/journal.pone.0230987

**Published:** 2020-03-24

**Authors:** Muhammad Ali Nawaz, Alice Valentini, Noor Kamal Khan, Christian Miquel, Pierre Taberlet, Jon E. Swenson

There are errors in the author affiliations. The correct affiliations are as follows:

Muhammad Ali Nawaz^1,2,3^, Alice Valentini^5,6^, Noor Kamal Khan^3^, Christian Miquel^5^, Pierre Taberlet^5^, Jon E. Swenson^2,4^

**1** Department of Animal Sciences, Quaid-i-Azam University, Islamabad, Pakistan, **2** Department of Ecology and Natural Resource Management, Norwegian University of Life Sciences, Norway, **3** Himalayan Wildlife Foundation, Islamabad, Pakistan, **4** Norwegian Institute for Nature Research, Trondheim, Norway, **5** Laboratoire d’Ecologie Alpine, Universite´ Joseph Fourier, France, **6** Dipartimento di Ecologia e Sviluppo Economico Sostenibile, Università degli Studi della Tuscia, Viterbo, Italy.
